# Pectin-Based Films with Cocoa Bean Shell Waste Extract and ZnO/Zn-NPs with Enhanced Oxygen Barrier, Ultraviolet Screen and Photocatalytic Properties

**DOI:** 10.3390/foods9111572

**Published:** 2020-10-29

**Authors:** Ana Cristina Mellinas, Alfonso Jiménez, María Carmen Garrigós

**Affiliations:** Department of Analytical Chemistry, Nutrition & Food Sciences, University of Alicante, San Vicente del Raspeig, ES-03690 Alicante, Spain; cristina.mellinas@ua.es (A.C.M.); alfjimenez@ua.es (A.J.)

**Keywords:** pectin, cocoa bean shell waste, zinc oxide nanoparticles, photocatalytic activity

## Abstract

In this work, pectin-based active films with a cocoa bean shell extract, obtained after waste valorisation of residues coming from the chocolate production process, and zinc oxide/zinc nanoparticles (ZnO/Zn-NPs) at different concentrations, were obtained by casting. The effect of the active additive incorporation on the thermal, barrier, structural, morphological and optical properties was investigated. Moreover, the photocatalytic properties of the obtained films based on the decomposition of methylene blue (MB) in aqueous solution at room temperature were also studied. A significant increase in thermal and oxidative stability was obtained with the incorporation of 3 wt% of ZnO/Zn-NPs compared to the control film. The addition of 5 wt% cocoa bean shell extract to pectin significantly affected the oxygen barrier properties due to a plasticizing effect. In contrast, the addition of ZnO/Zn-NPs at 1 wt% to pectin caused a decrease in oxygen transmission rate per film thickness (OTR.e) values of approximately 50% compared to the control film, resulting in an enhanced protection against oxidation for food preservation. The optical properties were highly influenced by the incorporation of the natural extract but this effect was mitigated when nanoparticles were also incorporated into pectin-based films. The addition of the extract and nanoparticles resulted in a clear improvement (by 98%) in UV barrier properties, which could be important for packaged food sensitive to UV radiation. Finally, the photocatalytic activity of the developed films containing nanoparticles was demonstrated, showing photodegradation efficiency values of nearly 90% after 60 min at 3 wt% of ZnO/Zn-NPs loading. In conclusion, the obtained pectin-based bionanocomposites with cocoa bean shell waste extract and zinc oxide/zinc nanoparticles showed great potential to be used as active packaging for food preservation.

## 1. Introduction

The use of biopolymers in films for food packaging has emerged as an alternative to plastic commodities by their bio-based origin, biodegradable character and possibilities to host multifunctional active agents [1]. In particular, the formulation of edible films obtained from lipids, proteins and polysaccharides as environmentally friendly materials for food packaging has been reported in the last few years [2,3,4]. Among them, polysaccharides are widely used in this application due to their large availability in nature, easy processing, cost-effectiveness and excellent biocompatibility [5]. The development of edible films based on polysaccharides has been reported to show good mechanical properties as well as environmentally friendly disposal after use. Biofilms based on cellulose [6] and derivatives [7], starch [8], chitosan [9,10] and pectin [11,12] have been proposed for food packaging applications.

Pectin is considered one of the most complex macromolecules in nature and it is formed by a group of structural polysaccharides, mostly containing galacturonic acid units [13]. Pectin is present in the primary cell walls of many plants, contributing rigidity to their structure, and it is frequently combined with lignin, hemicelluloses or cellulose. Pectin is basically composed of α-(1,4)-linked d-galacturonic acid [14] and its properties are influenced by the methyl esterification degree, which depends on the plant origin and the processing conditions [15]. In general, pectin shows good biodegradation performance, biocompatibility and non-toxicity, making it a good biomaterial for applications such as pharmaceutics, food packaging and cosmetics [16,17,18]. Nevertheless, pectin-based films also show some shortcomings, such as their poor barrier properties to water and gases [19,20]. The incorporation of hydrophobic compounds, such as essential oils, to the polymer matrix [19] or the development of blends using other polymers, such as chitosan [2,21,22], polyethylene glycol [23] and natural proteins [24,25], have been proposed as adequate strategies to improve the barrier properties of neat pectin. Moreover, the reinforcement with nanomaterials to produce pectin-based nano-biocomposites with improved properties has been recently proposed [26,27,28,29].

The use of sustainable and functional food packaging materials with natural additives or extracts has been proposed to improve the packaging functionalities as well as food quality and safety [30,31,32]. These functional additives are essential in the reduction or even complete elimination of the main food spoilage causes, such as rancidity, colour loss/change, nutrient losses, dehydration, microbial proliferation and off-odour production [33]. In this context, cocoa bean shell (CBS) is the major by-product obtained from the cocoa bean during the processing of chocolate and other food products [34]. CBS extracts could be a rich source of bioactive compounds, including polysaccharides, proteins and phenolic compounds, with high potential antioxidant/antimicrobial properties [35].

The interesting functionalities of zinc oxide nanoparticles (ZnO-NPs) such as antibacterial, antifungal, UV filtering properties, high catalytic and photochemical activities and non-toxicity to the environment have been reported by some authors [36,37]. In addition, these nanoparticles have been recently approved by the European Food Safety Authority (EFSA) to be used as a transparent ultraviolet (UV) light absorber in polyolefins for food packaging applications [38]. The addition of ZnO-NPs to polymer matrices such as polyurethane and chitosan has been reported to decrease water vapor and oxygen transmission rate compared to the neat films [39] while some authors reported the use of ZnO-NPs in modified atmosphere packaging (MAP) [40]. Few reports have been found combining ZnO-NPs and pectin. The development of chitosan/pectin/ZnO porous films for wound healing [41] and pectin/alginate/ZnO-NPs films as active packaging for food preservation [42] has been recently reported. Moreover, pectin-based gels with ZnO-NPs have been prepared for drug delivery applications [43] and as adsorbents for cationic dyes [44]. This work reports for the first time the development and characterisation of pectin-based active films with ZnO/Zn-NPs and a cocoa bean shell extract (CBSE) obtained from food by-products. The effect of these additives at different content levels was evaluated by studying the structural, morphological, barrier, thermal, optical and photocatalytic properties of the developed films.

## 2. Materials and Methods

### 2.1. Materials and Reagents

Cocoa bean shell residue was obtained as a by-product from a local chocolate producer. Pectin from citrus peel (galacturonic acid, ≥74%) and all other chemicals and reagents were purchased from Sigma-Aldrich (Madrid, Spain) and they were of analytical grade.

### 2.2. CBS Extract Preparation

Microwave-assisted extraction (MAE) was used to obtain CBS extracts from cocoa bean shell wastes by following a previously reported method [45]. The aim of this procedure was to obtain valuable fractions rich in active compounds (mainly polyphenols and polysaccharides) to be further used in the preparation of the pectin-based films. Prior to extraction, CBS was mixed with distilled water (0.04 g/mL) in a round-bottom flask and pH was adjusted to 2 with hydrochloric acid (1 M) and to 12 using sodium hydroxide (1 M) to obtain CBSE-2 and CBSE-12 extracts, respectively. Samples were subjected to extraction by using microwave equipment (Flexiwave, Milestone srl, Sorisole, Italy) at 100 °C for 5 min. The obtained mixture was then centrifuged for 20 min at 5300 rpm and 4 °C to recover the active compounds. The solid was separated and the different extracts were stored at −20 °C until further use.

### 2.3. Synthesis of ZnO/Zn-NPs

For the synthesis of ZnO/Zn-NPs, a previously optimised method was followed. Ten milliliters of a zinc chloride aqueous solution (0.37 M) were mixed with 50 mL of CBSE-12 solution (0.01 g/mL) and this mixture was kept under constant stirring (400 rpm). This solution was submitted to microwave heating (Flexiwave, Milestone srl, Sorisole, Italy) at 100 °C for 5 min and further centrifuged for 15 min at 5300 rpm and 4 °C. Finally, the solid was recuperated and calcined at 550 °C in a muffle furnace (JP Selecta, Barcelona, Spain) for 2 h and stored in darkness under vacuum conditions until use.

### 2.4. Pectin-Based Film Preparation

The casting technique was used for the development of pectin-based films. One gram of pectin was dissolved in distilled water (50 mL) under stirring at 70 °C. After complete dissolution, sorbitol (0.2 wt%) was added as plasticiser and pH was set to 4.5 with HCl 1 M. This mixture was stirred for 1 h at 400 rpm. Then, different concentrations of ZnO/Zn-NPs (1 and 3 wt%) were dispersed in 30 mL of distilled water and they were further sonicated for 30 min. A CBSE-2-based solution (0.05 wt%) was also prepared and added to the pectin solution, with or without ZnO/Zn-NPs, and sonicated for an additional 30 min. All solutions were finally casted into Petri dishes (15 cm diameter) and dried at 25 °C and 40% relative humidity in a climatic chamber (Dycometal, Barcelona, Spain) for 48 h. A control pectin film (PC) without the addition of any active additive was also prepared. The obtained formulations used in this work are presented in Table 1.

### 2.5. Pectin-Based Film Characterisation

#### 2.5.1. Moisture Content (MC)

The MC of pectin-based films was determined, in triplicate, according to the method proposed by Younis and Zhao [46]. Film samples were first conditioned in a desiccator containing CaCl_2_ at 10% relative humidity (RH) for 2 days, cut into 2 × 2 cm^2^ pieces and weighed (*W_i_*). Then, films were dried at 105 °C for 24 h and weighed again (*W_f_*). The MC was calculated according to Equation (1):

(1) $$MC\;\left(\%\right)=\left(\frac{W_{f}-W_{i}}{W_{i}}\right)\times 100$$

Thermogravimetric analysis (TGA) tests were performed, in triplicate, with a TGA/SDTA 851 Mettler Toledo thermal analyser (Schwarzenbach, Switzerland). Approximately 6 mg of each sample were heated from 25 to 700 °C at 10 °C/min under a nitrogen atmosphere (flow rate 50 mL/min).

Differential scanning calorimetry (DSC) tests were carried out to determine glass transition temperature (T_g_) in all pectin-based films by using a TA DSC Q-2000 instrument (New Castle, DE, USA) under a nitrogen atmosphere (flow rate 50 mL/min). Four-milligram samples were initially submitted to −90 °C in isothermal mode for 3 min. The temperature program followed consisted of a first heating from −90 to 150 °C, then cooling to −90 °C and a further second heating to 150 °C, with all these stages at a 10 °C/min heating/cooling rate. Three replicates of each sample were performed.

The oxidation onset temperature (OOT) was also determined, in triplicate, by DSC in order to evaluate the oxidative stability of pectin-based films. Samples were heated up at 10 °C/min under a pure oxygen atmosphere (50 mL/min) from 25 °C up to the observation of the exothermic oxidation peak. OOT was calculated as the temperature for the intersection between the DSC baseline and the slope of the exothermic peak in each case.

#### 2.5.2. Oxygen Transmission Rate (OTR)

An oxygen permeation analyser (8500 model Systech, Metrotec S.A, Spain) was used for OTR tests. Pure oxygen (99.9%) was introduced into the upper half of the diffusion chamber while nitrogen was injected into the lower half, where an oxygen sensor was located. Films were cut into 14 cm diameter circles for each formulation and they were clamped in the diffusion chamber at 25 °C before testing. In order to calculate the oxygen transmission rate per film thickness (OTR.e) values, the thickness of films at 10 different positions was measured with a digital micrometer (±0.001 mm, Mitutoyo, Japan). Tests were performed in triplicate.

#### 2.5.3. Optical Properties

Ultraviolet–visible (UV–Vis) spectra of pectin-based films were obtained, in triplicate, with a Biomate-3 Spectrophotometer (Thermospectronic, Mobile, AL, USA). Film pieces, 10 × 30 mm^2^, were placed on the internal side of the spectrophotometer cells and transmittance values at 280 and 600 nm were used to evaluate their optical properties.

Colour modifications on pectin-based films caused by the addition of the active additives were studied by using a VIS Minolta CM-2600d portable reflection spectrophotometer. Colour values were expressed as L* (lightness), a* (red/green) and b* (yellow/blue) coordinates in the CIELab colour space. These parameters were determined at five random different locations around the films’ surface and the average values were calculated. Since samples were transparent, these measurements were taken over a white background. Total colour difference (Δ*E*) was calculated according to Equation (2).

(2) $$\Delta E=\sqrt{{\left(L_{i}^{*}-L\right)}^{2}+{\left(a_{i}^{*}-a\right)}^{2}+{\left(b_{i}^{*}-b\right)}^{2}}$$

where *L*, *a* and *b* correspond to the coordinates of the control sample (pure pectin film) and *L_i_*, *a_i_* and *b_i_* are the coordinates of each different formulation.

#### 2.5.4. Attenuated Total Reflectance–Fourier Transform Infrared Spectroscopy (ATR–FTIR)

FTIR spectra of all film samples were recorded with a Bruker Analitik IFS 66/S (Ettlingen, Germany) infrared spectrophotometer equipped with a KBr beam splitter, a deuterated triglycine sulphate (DTGS) detector and Bruker OPUS software (Version 3.1). The analysis was performed in triplicate in the attenuated total reflectance (ATR) mode by using a Golden Gate accessory with a diamond crystal. Absorption spectra were obtained in the 4000–500 cm^−1^ range using 64 scans and a resolution of 4 cm^−1^.

#### 2.5.5. X-ray Diffraction (XRD)

XRD patterns of pectin-based films were recorded on a Bruker (Billerica, MA, USA) D8-Advance diffractometer equipped with a Goebel mirror for non-planar samples, a high-temperature chamber (up to 900 °C), a Kristalloflex K 760-80F X-ray generator (power 3000 W, voltage 20–60 kV and current 5–80 mA) and an X-Ray tube with a copper anode. Data were recorded by using Cu Kα radiation (1.5406 Å).

#### 2.5.6. Field Emission Scanning Electron Microscopy (FESEM)

The surface and the cross-section of all pectin-based films were studied by FESEM (Supra 25-Zeiss, Jena, Germany) in order to evaluate their homogeneity and the influence of the addition of the active additives on the polymer morphology. Samples were coated with a gold layer prior to analysis in order to increase their electrical conductivity.

#### 2.5.7. Photocatalytic Activity

The photocatalytic activity of the pectin-based films was evaluated by following a method based on the decomposition of methylene blue (MB) in aqueous solution at room temperature [8]. In brief, 50 mg of films were suspended in a beaker containing 50 mL of a 15 mg/L MB aqueous solution. Prior to irradiation, these suspensions were sonicated in the dark for 30 min to achieve the maximum adsorption of the dye onto the film surface. Then, the suspension was horizontally irradiated with a UV lamp under constant orbital stirring. After 60 min of irradiation, 5 mL of the dye solution were taken and the MB concentration in the solution was determined by UV–Vis spectroscopy at 664 nm. Tests were performed in triplicate for each formulation. The degradation efficiency (%) of MB was calculated with Equation (3):

(3) $$Degradation\;efficiency\;\%=\left(\frac{C_{i}-C_{t}}{C_{i}}\right)\times 100$$

where *C_i_* is the initial MB concentration (15 mg/L) and *C_t_* is the dye concentration at time *t* (mg/L).

### 2.6. Statistical Analysis

Statistical analysis of results was performed with the Statgraphics Centurion XVI statistical software. An analysis of variance (ANOVA) was carried out. Differences between values were assessed based on confidence intervals by using Tukey’s test at a *p* ≤ 0.05 significance level.

## 3. Results

### 3.1. Moisture Content

The moisture content determined in pectin-based films was related to the amount of water adsorbed onto their surface [47] and the obtained results are shown in Table 1. Nanocomposite active films exhibited a slightly lower moisture content although no significant differences were obtained compared to the neat pectin film (*p* > 0.05). This slight decrease in moisture content could be due to the formation of hydrogen bonds between the additives and the pectin structure which could reduce the diffusion of water molecules through the material [48].

### 3.2. Thermal Properties

**Figure 1:**
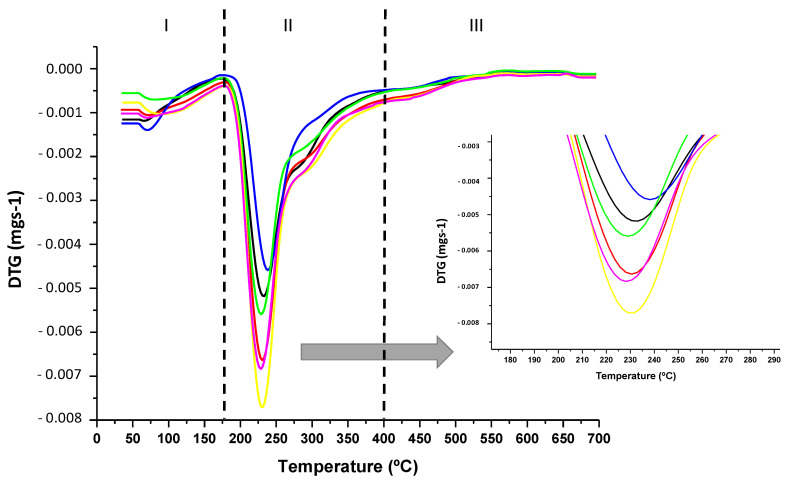
DTG curves of pectin-based films: PC (------), PC-1ZnO (------), PC-3ZnO (------), PC-5E (------), PC-5E-1ZnO (------) and PC-5E-3ZnO (------).

TGA and DSC tests were performed to study the influence of the addition of ZnO/Zn-NPs and CBSE-2 on the thermal properties of the obtained films. The most relevant results are shown in Table 2. The thermograms obtained for all formulations showed three different regions, as can be observed in the differential thermogravimetric (DTG) curves (Figure 1). The first thermal step, in the 50–150 °C range, was attributed to the desorption of water linked to hydrophilic groups in the pectin structure [19,23,49]. The second degradation step was observed between 200 and 400 °C, corresponding to the main thermal degradation of pectin. For the neat polymer, the maximum degradation peak was observed at 231 ± 1 °C (Table 2), as expected from the decomposition of galacturonic acid chains followed by the decarboxylation of the ring and acid side groups, with the formation of several low molecular weight gases and solid char [49,50]. Finally, slight weight loss at temperatures higher than 400 °C was observed in all formulations due to the degradation of the organic matter and C–C bonds remaining after the pyrolysis process [51].

All composite films showed a similar behaviour in their thermal degradation profile, suggesting that the addition of the active compounds did not significantly influence the pectin thermal degradation under a nitrogen atmosphere. However, in the case of the PC-3ZnO film, a significant increase (*p* < 0.05) in the maximum degradation temperature (239 ± 2 °C, Table 2) was observed (see zoomed-in area in Figure 1) which could be attributed to the strong interaction between the ZnO/Zn-NPs and the pectin structure via hydrogen bonding, as has been recently reported in other composites with zinc oxide nanoparticles and biopolymer matrices [52], resulting in a significant increase in the thermal stability in this formulation [53]. In addition, it should be noted that, due to the high thermal conductivity of ZnO/Zn-NPs, they could act as heat barriers, facilitating dissipation in the inner part of the composite matrix and likewise resulting in an improvement in the thermal stability of the overall nanocomposite, as has been recently reported [54].

Glass transition temperatures (T_g_) were also obtained for all films and results are shown in Table 2. T_g_ depends on the polymer structural arrangement and gives an indication of the torsion oscillation of the carbon backbone in the polymers’ structure [55]. No significant differences (*p* > 0.05) between the different active films and neat pectin were observed, suggesting that the addition of ZnO/Zn-NPs and CBSE-2 did not result in significant changes in the intrinsic pectin structure. A similar behaviour was observed by other authors in different polymer matrices after the incorporation of natural extracts and nanoparticles [11,56,57].

OOT measurements represent a very useful tool to evaluate the effect of the addition of different additives in the resistance of a polymer material to oxidative degradation processes [58]. The OOT values obtained for all pectin-based films are also shown in Table 2. An increase of around 10 °C was obtained with the incorporation of 3 wt% of ZnO/Zn-NPs compared to the control film, suggesting that the addition of these nanoparticles allows the thermo-oxidative stability of the polymer matrix to be increased. This behaviour is in agreement with the significant (*p* < 0.05) increase in thermal resistance observed for this formulation by TGA. Lower significant differences (*p* < 0.05) in OOT values (around 4 °C) were also observed between neat poly(ε-caprolactone) (PCL) and PC-5E, PC-5E-1ZnO and PC-5E-3ZnO films. These results can be explained by the presence of some antioxidant compounds in CBSE-2, mainly theobromine, caffeic acid, epicatechin and protocatechuic acid, showing that this extract has a high antioxidant capacity according to previous work [35,45], and reported free radical scavenging activity of zinc oxide nanoparticles [59]. Some authors have already reported the antioxidant effect of ZnO/Zn-NPs and they have justified this activity against oxygen by the large number of active sites present in these nanoparticles, which are able to fix oxygen molecules to their surface, with the consequent reduction of the oxidation reactions [60]. This effect could be really important for the potential application of these films for food packaging when the packaged food could suffer oxidative degradation. It has also been reported that the combination of Zn-based nanoparticles and some other compounds with well-known antioxidant activity could result in synergistic effects with an increase in the resistance to oxygen-induced damage in packaged food [61]. The positive effect of the addition of antioxidant compounds on the increase in OOT values has been previously reported in other polymer matrices, such as poly(lactic acid) (PLA) [62], poly(hydroxybutyrate) (PHB) [63] and polypropylene (PP) [64]. All these authors attributed this increase in OOT values to the prevention of the formation of free radicals caused by the chemical reactions with antioxidant compounds, delaying in this way the overall material oxidation.

### 3.3. Oxygen Transmission Rate (OTR)

The determination of barrier properties in films intended to be used in food packaging is essential to extend the food shelf-life by preventing humidity or diffusion of oxygen, ethylene, aroma or undesired flavours [22] which could produce adverse reactions and modify the organoleptic properties and/or quality of the food product [65]. Table 1 shows the results obtained for OTR.e in all pectin-based films. Significant differences (*p* < 0.05) were found between PC and PC-5E films, which were related to a plasticising effect due to the incorporation of active compounds in CBSE-2 into the pectin matrix, facilitating the diffusional process of oxygen molecules through the polymer matrix, as already reported for similar biopolymer matrices [66]. The addition of the active extract could contribute to an increase in the molecular mobility and the formation of a less packed network structure, separating pectin chains by creating voids and channels for oxygen molecules to pass through the polymer matrix, resulting in a higher oxygen transmission in the PC-5E formulation, as already reported by other authors [67,68].

In contrast, the addition of ZnO/Zn-NPs at 1 wt% to pectin significantly improved (*p* < 0.05) the oxygen barrier in all cases, causing a decrease in OTR.e values of approximately 50% compared to the control film (Table 1). This effect was associated with the formation of hydrogen bonds between ZnO/Zn-NPs and the pectin matrix, resulting in a decrease in the diffusion of oxygen molecules by producing a tortuous pathway through the films’ structure [69,70]. Similar results were obtained for pectin-based films after the incorporation of montmorillonite [71] and carbon nanotubes [72]. However, when the concentration of ZnO/Zn-NPs was increased up to 3 wt%, the expected improvement in oxygen barrier properties was not observed, with similar OTR.e values not significantly differing (*p* > 0.05) from those obtained for the control film (Table 1). Although it is supposed that the formation of hydrogen bonds would be more favoured by the addition of larger ZnO/Zn-NPs amounts, high concentrations of these nanoparticles could result in some agglomerations, generating holes inside the nanocomposite structure that would allow an easier diffusion of oxygen [57,73]. Finally, a decrease in OTR.e value was observed for the PC-5E-3ZnO film, which might be due to the improvement in the dispersion of ZnO/Zn-NPs in the polymer matrix after the addition of CBSE-2, as already reported for other metallic nanoparticles [74], although no significant differences (*p* > 0.05) in OTR.e values were observed with the control film.

### 3.4. Optical Properties

**Figure 2:**
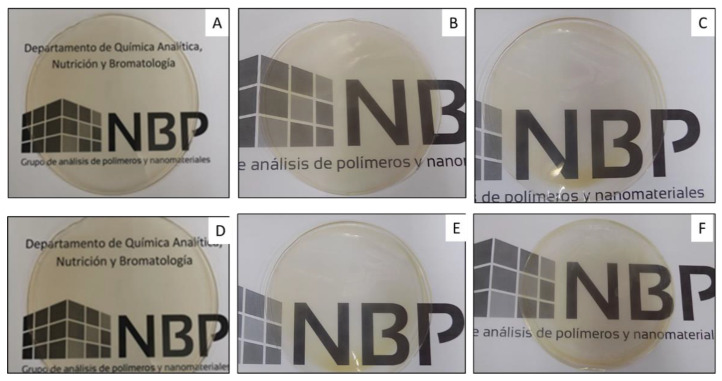
Visual appearance of pectin-based films: (**A**) PC, (**B**) PC-1ZnO, (**C**) PC-3ZnO, (**D**) PC-5E, (**E**) PC-5E-1ZnO and (**F**) PC-5E-3ZnO.

The visual appearance of films for food packaging applications is a critical issue in most cases and the addition of compounds that could be structurally bound to the polymer matrix could result in changes in the optical properties of the resulting films. In this study, all pectin-based films were visually transparent (Figure 2), smooth surfaced, homogeneous and flexible. The CIELab colour parameters (L*, a*, b*) and total colour differences (Δ*E*) obtained for pectin-based films are shown in Table 3. As can be observed, the b* parameter, which is related to blue–yellow colour changes, was not significantly affected (*p* > 0.05) by the incorporation of the active compounds. Regarding L* and a* parameters, no significant differences (*p* > 0.05) between PC, PC-1ZnO and PC-3ZnO films were found. However, significant differences (*p* < 0.05) were observed in these parameters with the incorporation of CBSE-2 in pectin-based films. A significant increase (*p* < 0.05) in a* values due to the intrinsic reddish colour of the cocoa extract was obtained. Moreover, the addition of ZnO/Zn-NPs to active films containing CBSE-2 produced a general significant decrease (*p* < 0.05) in a* values, which was more apparent at high concentrations of the nanoparticles, which might be due to the whitish coloration of ZnO/Zn-NPs [75]. Lightness was also affected by the incorporation of CBSE-2 in pectin-based films, with a significant decrease (*p* < 0.05) in L* values. The addition of ZnO/Zn-NPs and CBSE-2 (PC-5E-1ZnO and PC-5E-3ZnO formulations) resulted in some lower but not significant (*p* > 0.05) L* values compared to the control film. A similar behaviour was recently reported for pectin-based nanocomposite films with the addition of active compounds, contributing to noticeable colour changes in the developed films [76].

The developed pectin-based films were analysed by using UV–Vis spectrophotometry at 660 nm and 280 nm in order to evaluate transparency and UV blocking ability, respectively. High transparency values were obtained (Table 3) for all pectin-based films with transmittance values higher than 75%, considering the obtained films as optically clear [68], although a significant decrease (*p* < 0.05) was observed with ZnO/Zn-NPs with the addition of CBSE-2. These differences in transparency found between the control and active films may be due to the formation of more compact films by the interaction of the pectin matrix with the active compounds present in CBSE-2, mainly polyphenols [45], and the presence of ZnO/Zn-NPs contributing to the reduction of light intensity passing through the films [77]. The incorporation of ZnO/Zn-NPs also increases the amount of inorganic material not homogeneously distributed in the polymer matrix, resulting in a reduction in transparency [70].

Transmittance values obtained at 280 nm for control films (25 ± 2%, Table 3) indicated that neat pectin was a fairly good UV barrier by absorbing around 75% of the incident UV radiation [56]. A significant improvement (*p* < 0.05) in UV barrier properties was obtained for the active films (Table 3) with the addition of CBSE-2 and ZnO/Zn-NPs, with about 98% of the UV radiation screened by these films. This behaviour was related to the presence of phenolic compounds in cocoa extracts [78] and the studied nanoparticles [57,79]. In conclusion, the addition of CBSE-2 and ZnO/Zn-NPs to pectin-based films has shown great potential for UV light prevention to avoid photocatalytic reactions in packaged food.

### 3.5. ATR-FTIR Analysis

**Figure 3:**
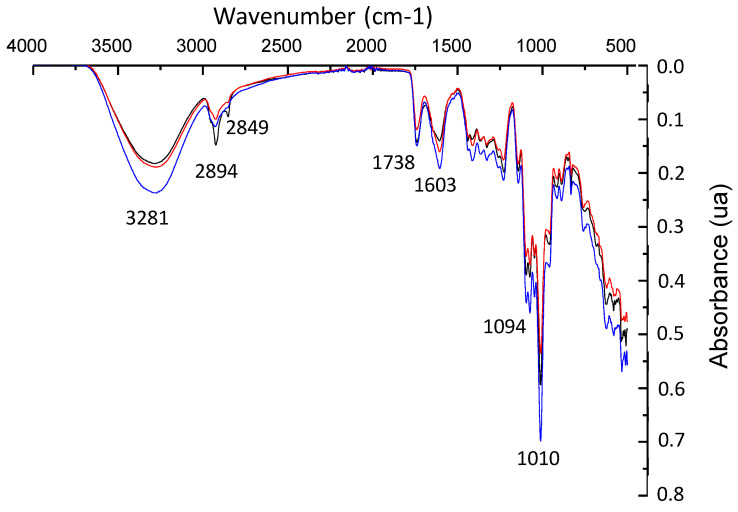
FTIR spectra of PC (------), PC-1ZnO (------) and PC-3ZnO (------) films.

The FTIR spectra of neat pectin, CBSE-2 and active pectin-based films exhibited distinctive peaks in the 4000–500 cm^−1^ range. The PC film (Figure 3) showed a broad peak centred at 3281 cm^−1^ which was attributed to O–H stretching vibrations [11], while bands centred at 2894 and 2849 cm^−1^ were related to C–H stretching vibrations of methylene groups and methyl group of pectin polymer chains, respectively [80]. The intense bands observed at 1738 and 1603 cm^−1^ were associated with the ester stretching vibrations of the -COCH_3_ group and the asymmetric stretching vibrations of the carboxylate anion –COO^−^, giving an indication of the high methoxylation of the pectin molecules [19,81]. Some other bands were also obtained in the 1360–800 cm^−1^ range, which were related to the stretching vibrations of the C–O–C and C–C bonds of the carbohydrate ring [82].

Figure 3 also shows the FTIR spectra obtained for PC-1ZnO and PC-3ZnO films. The addition of ZnO/Zn-NPs did not produce significant changes in the structure of the polymer matrix, but a decrease in the intensity of the 2894–2849 cm^−1^ bands at both nanoparticle concentrations was observed, which was related to the formation of hydrogen bonds between ZnO/Zn-NPs and the pectin matrix. A significant shift (3256 cm^−1^) and increase in intensity of the band related to O-H stretching vibrations was also shown at 3 wt% of ZnO/Zn-NPs in PC-3ZnO films [43].

**Figure 4:**
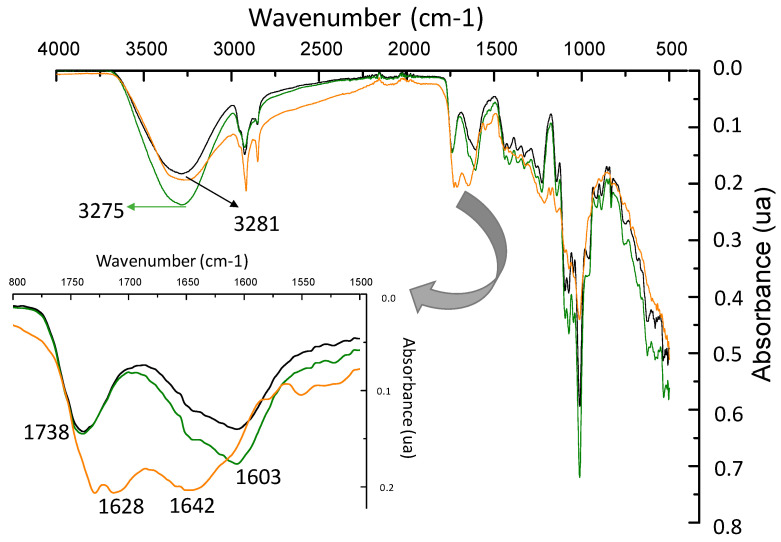
FTIR spectra of PC (------) and PC-5E (------) films and CBSE-2 (------).

The FTIR spectra of PC and PC-5E films and CBSE-2 are compared in Figure 4. As can be seen, no important changes in the spectrum of neat pectin were observed with the addition of 5 wt% of CBSE-2. However, a significant shift of the band at 3275 cm^−1^ was found in the PC-5E film compared to the CBSE-2 spectrum, which was associated with the interaction between the hydroxyl groups of the polyphenolic compounds and polysaccharides present in the cocoa extract [83] with the pectin matrix. The typical absorption peaks of polysaccharides at 1014, 1070 and 1119 cm^−1^ were observed in the CBSE-2 spectrum and they were assigned to the –C–OH, –C–C– and –C–O– vibration modes, respectively [84]. Finally, two bands centred at 1628 and 1642 cm^−1^ with similar intensity were detected in CBSE-2 spectra, confirming the presence of some pectin in CBSE-2, as these bands are usually used to determine the esterification degree of pectin [83,85,86], suggesting a value around 50%.

**Figure 5:**
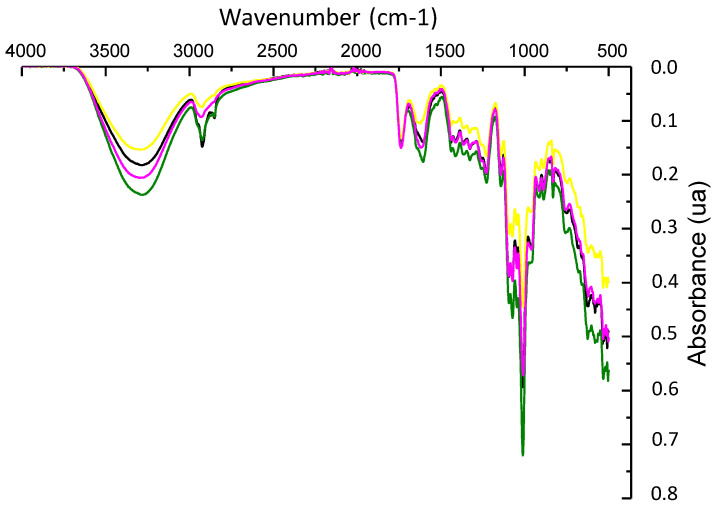
FTIR spectra of PC (------), PC-5E (------), PC-5E-1ZnO (------) and PC-5E-3ZnO (------) films.

Finally, Figure 5 shows the FTIR spectra obtained for PC, PC-5E, PC-5E-1ZnO and PC-5E-3ZnO films. No noticeable changes were obtained, suggesting that the addition of CBSE-2 and ZnO/Zn-NPs did not produce significant changes in the pectin structure. A similar behaviour was reported by other authors with the incorporation of ZnO-NPs [87] or natural extracts derived from plants [22] into polymer matrices.

### 3.6. X-ray Diffraction Analysis

**Figure 6:**
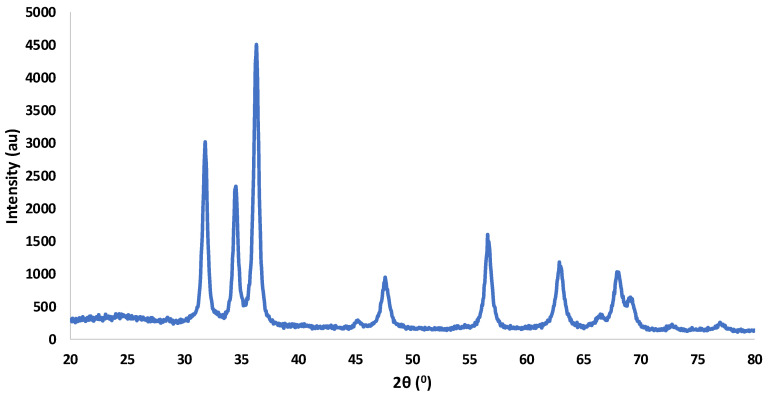
XRD pattern of ZnO/Zn-NPs.

The XRD pattern of ZnO/Zn-NPs is shown in Figure 6. Different characteristic diffraction peaks at 2θ = 31.8, 34.5, 36.3, 47.5, 56.6, 62.9, 66.5, 68.3, 69.1, 72.8 and 77.1° were observed. These peaks were ascribed, respectively, to the (1 0 0), (0 0 2), (1 0 1), (1 0 2), (1 1 0), (1 0 3), (2 0 2), (1 1 2), (2 0 1), (0 0 4) and (2 0 2) planes of hexagonal wurtzite structure ZnO according to JCPDS Card No. 79-0206. Similar planes were reported for ZnO-NPs by other authors [88,89,90]. Besides, these nanoparticles showed a small but noticeable peak at 46°, indicating the presence of interstitial zinc in the ZnO lattices [91].

**Figure 7:**
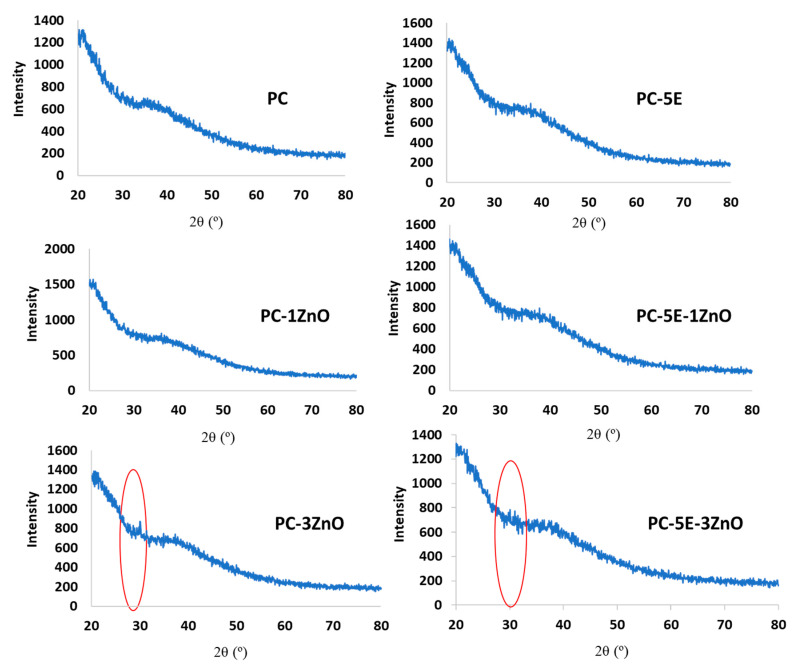
XRD patterns of pectin-based films.

Regarding pectin-based films, no specific diffraction peaks were observed for the neat pectin film (Figure 7), giving a clear indication of its amorphous structure as already reported by other authors [19,26,43]. The diffraction pattern of the studied nanoparticles was overcome by that of pectin at low ZnO/Zn-NP content [28], resulting in a completely amorphous structure for pectin-based films with 1 wt% of ZnO/Zn-NPs. However, when the concentration of ZnO/Zn-NPs was increased up to 3 wt%, two diffraction peaks between 30 and 35° were detected in films (circled area in Figure 7), corresponding to the major diffraction peaks observed in nanoparticles and suggesting the good incorporation of ZnO/Zn-NPs into the pectin matrix.

### 3.7. Morphological Analysis

**Figure 8:**
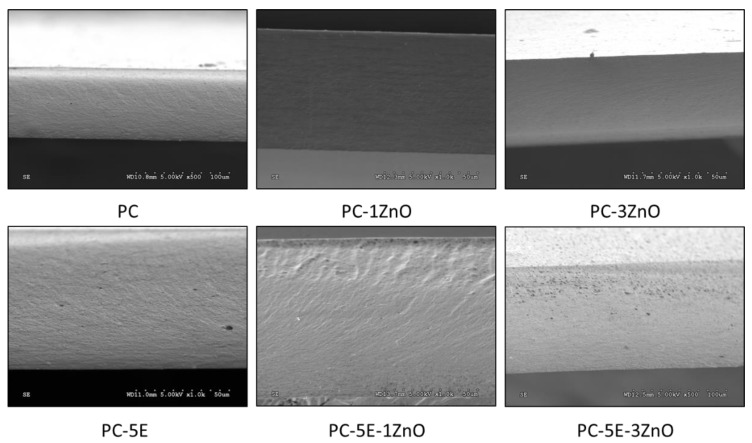
SEM micrographs of cross-sections of pectin-based films.

The microstructure of active pectin-based films containing CBSE-2 and ZnO/Zn-NPs was studied by SEM to get insight into the incorporation of the active compounds, their organisation along the biopolymer film matrix and their possible influence on the films’ final properties. The cross-section micrographs of all pectin-based films are shown in Figure 8. A uniform and homogeneous surface was observed in all samples, demonstrating the good incorporation of CBSE-2 and ZnO/Zn-NPs into the pectin matrix. In addition, PC5E-1ZnO and PC5E-3ZnO films showed an apparent improvement in the nanoparticle dispersion through the pectin matrix when compared to film samples without the incorporation of CBSE-2. This behaviour was related to the presence of some antioxidant compounds in CBSE-2, increasing the possibility of the formation of hydrogen bonds between active compounds, nanoparticles and the pectin matrix [55]. The presence of both active additives in pectin-based films also placed ZnO/Zn-NPs mainly on the surface of the pectin films, particularly at the highest nanoparticle concentration. This behaviour may be due to the higher affinity between pectin and CBSE-2, displacing the nanoparticles to the material surface [70].

### 3.8. Photocatalytic Properties

Methylene blue (MB) is a cationic-type model dye which is commonly used in medicine and paper and textile industries [92]. The mechanism of the photocatalytic degradation of MB with ZnO-NPs has been reported by other authors. When ZnO is exposed to irradiation, electron–hole pairs are created and afterwards superoxide radicals are formed by the reaction of electrons with oxygen and hydroxyl radicals generated by the reaction of holes with hydroxide ions/water, which results in the rupture of organic bonds and the degradation of the MB molecules [93,94,95]. In the case of CBSE-2, antioxidant compounds, such as epicatechin or caffeic acid, might produce hydroxyl radicals under irradiation able to degrade the MB molecules [96].

The results obtained for MB degradation with all pectin-based formulations are shown in Table 1. As can be seen, the neat pectin film did not produce any photocatalytic activity. However, a significant increase (*p* < 0.05) in photodegradation efficiency was obtained in formulations with nanoparticles, showing values of around 90% at 3 wt% of ZnO/Zn-NP loading (PC-3ZnO and PC-5E-3ZnO nanocomposites). The positive effect of ZnO/Zn-NPs in the photodegradation of MB in aqueous solution has already been demonstrated [91]. The presence of interstitial zinc in these nanoparticles enhanced their photocatalytic activity compared to ZnO-NPs by an increase in the lifetime of the electron–hole pairs, with this effect being more pronounced with increasing ZnO/Zn-NP content [94]. Similar results were obtained in starch/polyvinyl alcohol/TiO_2_-NP composite films after 90 min of testing [8].

## 4. Conclusions

CBSE-2 and ZnO/Zn-NPs were successfully added to pectin to obtain biodegradable films with no major changes in moisture content, structural, transparency and morphological properties. These additives showed high compatibility with the pectin matrix and their incorporation greatly improved oxygen, thermal and UV barrier properties, which are very important for the intended application of these biomaterials in food packaging. In particular, the barrier to oxygen was enhanced by 50%, while the screen to UV radiation reached 98%, which are essential properties for the packaging of food highly sensitive to oxidative degradation. Besides, high photocatalytic activity was also demonstrated in the nanocomposite films with the addition of ZnO/Zn-NPs, which was related to the presence of interstitial zinc in these nanoparticles. In conclusion, the pectin-based bionanocomposite films obtained in this work have shown great potential to be used in different fields, particularly in food packaging. Further specific studies on the developed biomaterials, such as mechanical performance and water absorption measurements, should be carried out to finally assess their suitability for this application. Moreover, the processes and materials proposed in this work could also contribute to the circular bioeconomy concept in the agriculture and food processing sectors by valorising cocoa bean shell residues and using biopolymers to obtain sustainable functional materials for food packaging and preservation.

## Figures and Tables

**Table 1:** Moisture, thickness, barrier properties and photocatalytic activity of pectin-based films.

Formulation	CBSE-2 (wt%)	ZnO/Zn-NPs (wt%)	Moisture (%)	Thickness * (mm)	OTR.e (cm^3^ mm m^−2^ day)	Degradation Efficiency (%)
PC (control)	-	-	18 ± 3 ^a^	0.087 ± 0.022 ^a^	0.51 ± 0.07 ^a^	4 ± 2 ^a^
PC-1ZnO	-	1	13 ± 1 ^a^	0.073 ± 0.031 ^a^	0.32 ± 0.02 ^b^	72 ± 7 ^b^
PC-3ZnO	-	3	14 ± 3 ^a^	0.070 ± 0.024 ^a^	0.51 ± 0.09 ^a^	87 ± 4 ^c^
PC-5E	5	-	13 ± 1 ^a^	0.078 ± 0.016 ^a^	0.63 ± 0.01 ^c^	39 ± 6 ^d^
PC-5E-1ZnO	5	1	17 ± 1 ^a^	0.071 ± 0.011 ^a^	0.36 ± 0.02 ^b^	84 ± 4 ^bc^
PC-5E-3ZnO	5	3	16 ± 4 ^a^	0.085 ± 0.015 ^a^	0.35 ± 0.13 ^ab^	88 ± 2 ^c^

Mean ± SD, *n* = 3 (* *n* = 10 for thickness). Different superscripts within the same column indicate significant differences between formulations (*p* < 0.05). CBSE-2: cocoa bean shell waste extract obtained at pH = 2. ZnO/Zn-NPs: zinc oxide/zinc nanoparticles. OTR.e: oxygen transmission rate per film thickness.

**Table 2:** Thermal properties of pectin-based films.

Formulation	T_max_ (°C)	T_g_ (°C)	OOT (°C)
PC	231 ± 1 ^a^	52 ± 1 ^a^	202 ± 1 ^a^
PC-1ZnO	229 ± 1 ^a^	51 ± 2 ^a^	202 ± 1 ^a^
PC-3ZnO	239 ± 2 ^b^	51 ± 1 ^a^	214 ± 2 ^b^
PC-5E	229 ± 1 ^a^	53 ± 2 ^a^	206 ± 1 ^c^
PC-5E-1ZnO	230 ± 1 ^a^	53 ± 1 ^a^	205 ± 1 ^c^
PC-5E-3ZnO	229 ± 1 ^a^	52 ± 1 ^a^	207 ± 2 ^c^

Mean ± SD, *n* = 3. Different superscripts within the same column indicate significant differences between formulations (*p* < 0.05). OOT. Oxygen onset temperature. T_max_. Temperature of maximum degradation rate. T_g_. Glass transition temperature.

**Table 3:** Colour parameters and transparency of pectin-based films.

Formulation	L*	a*	b*	Δ*E*	T_280_ (%)	T_660_ (%)
PC	85.4 ± 2.6 ^a^	10.1 ± 3.1 ^a^	3.5 ± 1.9 ^a^		25 ± 2 ^a^	87 ± 3 ^a^
PC-1ZnO	86.0 ± 2.6 ^a^	8.2 ± 0.6 ^a^	3.6 ± 2.0 ^a^	2.6 ± 0.6 ^a^	2 ± 1 ^b^	77 ± 4 ^b^
PC-3ZnO	85.4 ± 2.4 ^a^	9.8 ± 0.5 ^a^	3.7 ± 1.8 ^a^	2.8 ± 0.1 ^a^	2 ± 1 ^b^	77 ± 3 ^b^
PC-5E	80.6 ± 2.6 ^b^	20.0 ± 2.2 ^b^	3.0 ± 2.3 ^a^	10.3 ± 2.7 ^b^	3 ± 1 ^b^	74 ± 3 ^b^
PC-5E-1ZnO	82.4 ± 2.4 ^ab^	16.9 ± 0.7 ^c^	3.4 ± 1.8 ^a^	7.1 ± 0.9 ^ab^	1 ± 1 ^b^	75 ± 3 ^b^
PC-5E-3ZnO	83.9 ± 2.4 ^ab^	14.0 ± 0.4 ^d^	3.6 ± 1.8 ^a^	4.3 ± 0.6 ^ab^	1 ± 1 ^b^	77 ± 3 ^b^

Mean ± SD, *n* = 5. Different superscripts within the same column indicate significant differences between formulations (*p* < 0.05).
